# Long-range PCRs and next-generation sequencing to detect cytomegalovirus drug resistance-associated mutations

**DOI:** 10.1128/aac.00141-25

**Published:** 2025-06-25

**Authors:** Julien Andreani, Aurélie Truffot, Valentin Tilloy, Hugo Jardin, Matilda Lespinasse, Marie Usal, Sylvie Larrat, Patrice Morand, Julien Lupo, Sébastien Hantz, Sophie Alain, Raphaële Germi

**Affiliations:** 1 Univ. Grenoble Alpes, CNRS, CEA, IRIG IBS, Grenoble, France; 2 Virology Laboratory, Institut de Biologie et de Pathologie, Grenoble, France; 3 Laboratoire de Bactériologie-Virologie-Hygiène, CHU Limoges 36715 , Limoges, France; 4 INSERM, RESINFIT, Limoges, France; IrsiCaixa Institut de Recerca de la Sida, Barcelona, Spain

**Keywords:** cytomegalovirus, next-generation sequencing, long-range polymerase chain reaction, antiviral resistance

## Abstract

Cytomegalovirus (CMV) infections occur in 10%–40% of organ or stem cell transplant patients. Despite the low prevalence, CMV antiviral resistance has an important impact on patient outcomes. Guidelines for transplant recipients recommend that resistance should be suspected in cases of unchanged or increasing CMV viral loads after a minimum of 2 weeks of antiviral therapy at an appropriate dose or >6 weeks of ganciclovir exposure. Next-generation amplicon sequencing (NGS) makes it possible to directly target the genes involved in this resistance. Currently, six drugs are available, and six CMV genes (UL54-UL97-UL89-UL56-UL51-UL27 genes) can harbor mutations affecting drug efficacy. Here, we developed different primers targeting these six genes with long-range polymerase chain reaction (PCR). Based on clinical requirements, all genes or a subset could be sequenced in a single run using Oxford Nanopore technology and combined with an automatic bioinformatics pipeline to detect and report mutations. We utilized 46 blood samples, five external quality controls, and 10 mixes of two bacmids provided by the national reference center (CNR) Herpesvirus Limoges, each carrying distinct mutations. Assay performance (sensitivity, specificity, and accuracy) was evaluated through an interlaboratory exchange with CNR Herpesvirus. Long-range PCR combined with next-generation sequencing analysis enables earlier and more comprehensive discrimination of the double population and determines whether the detected single-nucleotide polymorphisms are present on single or multiple CMV strains. We developed a next-generation sequencing assay combined with eight long-range PCRs to sequence all genes involved in CMV antiviral resistance and to detect early low-frequency mutations.

## INTRODUCTION

Human cytomegalovirus (CMV) is a common opportunistic agent of infection following transplantation (1). Belonging to the *Herpesviridae* family, CMV possesses a linear genome of approximately 230 kbp in length, encoding more than 150 proteins (2–4). CMV infections occur in 10%–40% of solid organ or hematopoietic stem cell transplant recipients (5, 6) and constitute major causes of morbidity and mortality (7–11). Currently, six drugs are available for the treatment of CMV infections. Four of these are inhibitors of the viral DNA polymerase encoded by the *UL54* gene: ganciclovir (GCV) and its oral prodrug, valganciclovir (valGCV), are guanosine nucleoside analogues requiring phosphorylation by the viral phosphotransferase encoded by the *UL97* gene; cidofovir (CDF) is a cytidine monophosphate analog; and foscarnet (FOS) a pyrophosphate analog. Maribavir (MBV) is an inhibitor of viral *UL97*-phosphotransferase, and letermovir is an inhibitor of the viral terminase complex encoded by *UL56-UL89-UL51* genes (12, 13).

Refractory CMV infections may arise from multiple factors, such as excessive over-immunosuppression, impaired CMV-specific T-cell immunity, sub-therapeutic concentrations of antiviral drugs, or antiviral drug resistance due to resistance mutations in viral genes encoding proteins targeted by antiviral agents (12, 14). Genotypic or viral resistance testing is recommended when CMV DNAemia or disease persists after 2 weeks of full-dose antiviral therapy (10, 12, 15). In the transplant setting, the incidence of drug resistance mutations ranges from 5% to 12% (7, 10, 12, 16) and is associated with younger age, low GCV exposure, recipients’ negative serostatus, infection on valGCV prophylaxis, inadequate antiviral drug delivery, and haploidentical hematopoietic stem cell transplant (14, 15, 17). Genotypic resistant strains are associated with poor outcomes at 1 year post-transplantation (17).

Antiviral resistance is mainly characterized by sequencing the *UL97* gene, which can detect resistance-associated mutations to GCV and MBV, or the *UL54* gene, which detects resistance-associated mutations to CDF, FOS, and GCV. Chou et al. recommended sequencing *UL56*, *UL89*, and *UL51* genes, which encode subunits of the terminase complex and determine resistance to letermovir (18, 19), and sequencing the *UL27* gene, which encodes a protein involved in nuclear egress and is known to decrease MBV susceptibility (13).

More recently, some laboratories have replaced Sanger-based sequencing, the gold standard for detecting resistance mutations, by next-generation sequencing (NGS). Sanger sequencing exhibits several limitations: a read length limit close to 700 base pairs; an inability to detect single nucleotide polymorphisms (SNPs) at less than 20% and quantify viral subpopulations in a sample (20, 21); its time-consuming nature; and its sequencing of a single gene target per reaction, which increases costs depending on the number of targets. The robustness and adaptability of NGS-based methods in the clinical setting explain the growing interest in this technology among clinical laboratories (22). NGS offers several advantages, including increased sensitivity for identifying minor variants, improved cost-effectiveness through multiplexing targets in single-run sequencing, and enhanced characterization of genomic diversity (23). Several technologies and strategies are currently based on the production of multiple amplicons (24) or shotgun sequencing after specific amplification (22, 25). However, none uses long-range PCR.

This study describes the development of long-range PCRs using high-throughput laboratory tests and their overall performance for clinical application. This assay facilitates the sequencing, within a single run, of amplicons obtained from eight long-range PCRs amplifying full-length genes.

## MATERIALS AND METHODS

### Patient samples and controls

The accuracy, sensitivity, and specificity of our CMV NGS assay were evaluated utilizing (i) 46 whole blood samples from residual care retrospectively selected based on their CMV viral load (nucleic-acid extraction and qPCR: EMAG automated system and CMV R-gene kit (bioMérieux), amplification platform: LightCycler480II (Roche Diagnostics)); (ii) four CMV DNA extracts artificially diluted in EMAG elution buffer and used for sensitivity analysis; (iii) five DNA controls from Quality Control for Molecular Diagnostics (QCMD, Glasgow, UK); and (iv) 10 mixtures of two different bacmids (CNR Herpesvirus, Limoges, France). The reproducibility tests were conducted on a clinical sample with a viral load of 2500 UI/mL. All CMV genes of interest (*UL97*, *UL54*, *UL56*, *UL89, UL51,* and *UL27*) of this sample were extracted, amplified, and sequenced six times. The coefficient of variation of nucleotides for all positions for each gene was calculated. Results of one additional clinical sample will be detailed to explain the ability of our assay to detect double viral population.

The total DNA was extracted from 200 µL of whole blood using EMAG (BioMerieux, Marcy l’Etoile, France) and eluted in 50 µL of elution buffer. The extraction protocol was the same for viral load testing and for the target DNA for obtaining long-range PCR products.

### Long-range PCR design and amplification

Primers for long-range PCRs were home-designed to have Tm close to 60°C (Table 1) using primer-BLAST online with standard parameters (26) and AD169 strain NCBI accession number X17403.1 as the reference. *UL54* gene (~4,000 bp) and *UL89* gene amplifications were each based on two primer pairs. Rescue primers (Table S1), employed in the event of non-amplification with the primers described in Table 1, were designed and tested.

**TABLE 1:** Primer sequences (forward and reverse) home-designed for long-range PCRs of CMV genes

Gene target	Sequence primer (5’- > 3’)	Reference position^*a*^	Product length (BP)
*UL54* Part 1	ACGTGAGCGAGTCCTTTGAG	76,609–76,628	2,250
	CTGCCGCGATTTTATCCTGC	78,859–78,840	
*UL54* Part 2	TGATAGCGGCGTTAGGTGAC	78,768–78,787	2,130
	TTCTTTGGACGGACGGACTG	80,895–80,876	
*UL97*	AAGTTGCTGGACGCTCTCTC	139,927–139,946	2,948
	GATGCGGTAGGCGTAAGACA	142,874–142,855	
*UL56*	GCCCAGCAGATAAGTGGTGT	83,004–83,023	3,391
	CGTCGCATCATCAACAGCAG	86,394–86,375	
*UL89* exon 1	CCGCCTGTCTGTGTTTGTTG	132,168–132,187	2,162
	CTTCGTATACCAGGCCCGTC	134,324–134,310	
*UL89* exon 2	CTTCGGAGGTCCTTTGGGTC	136,667–136,686	2,080
	CCTCGGAGACCGAGAAATCG	138,745–138,726	
*UL27*	CCCAACTGAAAAGGTTGGCG	32,544–32,563	2,278
	ACGTCAGCGAGTACGTGTTT	34,821–34,802	
*UL51*	GACCGTGTCTGTCTTGAGCA	73,107–73,126	1,300
	CTCTGGTCGCAGTCCTGTTT	74,445–74,426	

^*a*^ CMV strain AD169 NCBI accession no. X17403.1.

Long-range PCRs were performed separately using for each 0.120 µM of each primer, 12 µL of NEB Q5 Hot Start High-Fidelity 2X Master Mix (New England Biolabs, USA), and 10 µL of DNA for a 25 µL final volume for each mixture.

The amplification program consisted of 2 minutes at 98°C for Taq polymerase activation, 45 cycles of denaturation (98°C for 30 seconds)/annealing (60°C for 30 seconds)/elongation (72°C for 4 minutes), and a final extension of 2 minutes at 72°C.

### Library preparation and NGS sequencing

The Oxford Nanopore Technology (ONT) protocol was modified as follows: 10 µL of each amplicon was added to 2.5 µL of barcodes provided in the rapid barcoding kit RBK.110.96 (Oxford Nanopore, Oxford, UK) and subsequently incubated at 30°C for 2 minutes, followed by inactivation at 80°C for 2 minutes. The total volume of each barcoded amplicon was all combined in a single pool and purified. The recommendations for ONT library preparation were then followed. For each run, a maximum of 20 barcodes per flow cell were loaded. Sequencing was conducted for 12 hours on a Gridion flowcell R10 (Oxford Nanopore) with the adaptive sampling mode using an enrichment strategy based on the CMV AD169 strain genome reference and fast basecalling.

### Bioinformatics analyses

**Fig 1:**
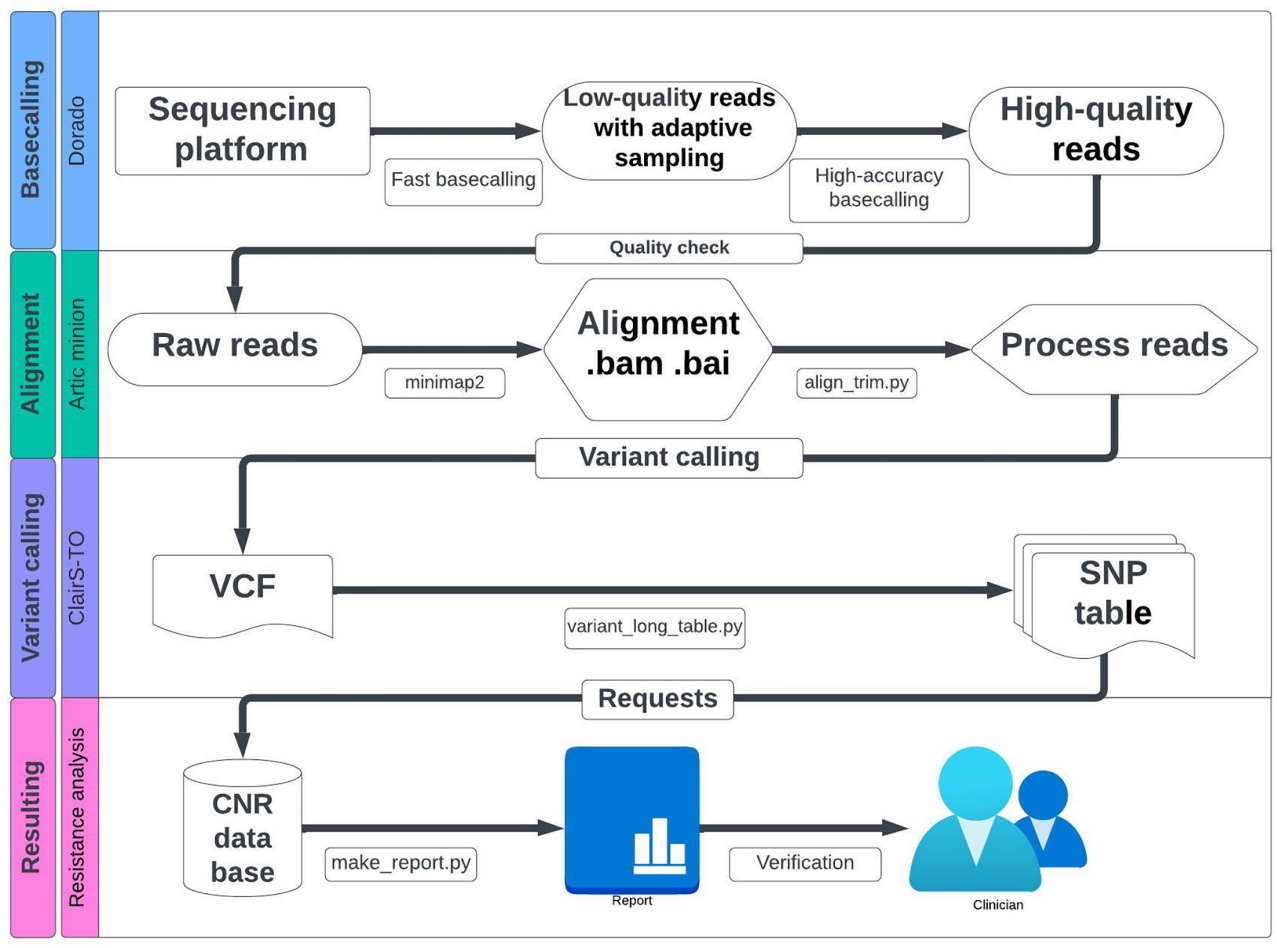
Flow chart of the Bioinformatics pipeline. Fast basecalling is first performed, followed by high-accuracy basecalling to provide higher raw read accuracy. Sequence mapping and alignment used minimap2, which realigns the remaining reads to the CMV AD169 genome reference (GenBank ID X17403.1). ClairS-TO generates the VCF. The list of SNPs obtained is compared with the CNR Herpesvirus, Limoges database (https://www.unilim.fr/cnr-herpesvirus/outils/codexmv/database/) to identify any mutations generating resistance. Finally, the generated report is validated by medical biologists and sent to clinicians. CNR: national reference center; SNP: single-nucleotide polymorphism; VCF: variant calling table.

Fast5 files were re-analyzed in high-accuracy basecalling to provide higher raw read accuracy. Fastq files were analyzed by using a modified pipeline adapted from nfcore/Viralrecon (27). Reads were mapped by minimap2 against the CMV AD169 genome reference, and consensus sequences were built. To determine minor variants, the variant call ClairS-TO was used. Finally, SNPs were compared with the French national reference database (CNR Herpesvirus; https://www.unilim.fr/cnr-herpesvirus/outils/codexmv/database/) (Fig. 1).

### Detection of minor variants

First, two DNA (DNA1 and DNA2) obtained from two CMV positive clinical samples presenting the same viral load (4 log IU/mL) but with different CMV strains were combined in different proportions. DNA1 and DNA2 were tested pure and mixed 50%/50%, 80%/20%, and 20%/80% in mixtures A, B, and C, respectively. For each, *UL54* and *UL97* genes were sequenced at the Grenoble Alpes University Hospital (CHUGA) using NGS-ONT and in CNR Herpesvirus Limoges using Sanger.

Second, two bacmids were produced by CNR Herpesvirus at 4 ng/mL (28). One bacmid had the E655D mutation on *UL97*, while the other had the N408S mutation on *UL54*. Bacmids were mixed in different ratios (100% to 2.5%), diluted tenfold to obtain 4 ng of DNA/µL, then amplified, and sequenced using CHUGA NGS-ONT technology and CNR Herpesvirus Sanger analysis.

## RESULTS

### Assay validation

#### Analytical sensitivity on clinical samples

The sensitivity of the CHUGA NGS ONT technology for each targeted *UL* gene was determined using different clinical samples with decreasing CMV DNA concentrations (Table 2). The overall performance of long-range PCR increased with increasing viral loads for each gene target. Sensitivity decreased below 2,600 IU/mL with variations depending on the primer pairs tested. PCR and sequencing of *UL89*-exon 1 exhibited the lowest sensitivity. A moderate negative correlation (linear regression, coefficient of correlation r^2^ = 0.53, *P* = 0.040) between the amplicon length and the frequency of successful amplification of targeted genes was observed. PCR and sequencing of *UL54*, *UL51*, and *UL27* showed superior performance.

**TABLE 2:** Sensitivity of CHUGA NGS technology for each targeted UL gene, represented by the frequency of success for amplification and sequencing

	Number of amplification and sequencing success for samples (% of success)							
IU/mL	UL97	UL54 part 1	UL54 part 2	UL56	UL89 exon 1	UL89 exon 2	UL51	UL27
0–1,290	2/5 (40)	0/4 (0)	1/5 (20)	2/4 (50)	0/3 (0)	0/3 (0)	4/5 (80)	3/5 (60)
1,291–2,600	4/18 (22.2)	7/14 (50)	3/8 (38)	0/11 (0)	2/8 (25)	3/8 (38)	6/6 (100)	6/6 (100)
2,601–3,900	17/25 (68)	18/20 (90)	12/15 (80)	14/20 (70)	6/14 (43)	11/15 (73)	Not tested	Not tested
>3,900	17/18 (94.4)	11/11 (100)	15/15 (100)	5/5 (100)	16/18 (89)	18/18 (100)	3/3 (100)	3/3 (100)
Overall performance	40/66 (60.6)	36/49 (73.5)	31/43 (72.1)	21/40 (52.5)	24/43 (55.8)	32/44 (72.7)	13/14 (92.9)	12/14 (85.7)
Performance up to 2,600 IU/mL^*a*^	34/43 (79)	29/31 (93)	27/30 (90)	19/25 (76)	22/32 (69)	29/33 (88)	3/3 (100)	3/3 (100)

^*a*^ Gray highlight shows that if we evaluate the CHUGA NGS ONT technology sensitivity only on samples with a viral load up to 2,600 UI/mL in whole blood, the performance is >75% for all the genes analysed.

#### Reproducibility

After six PCR and sequencing of all CMV genes of interest, the coefficients of variation for all genes were below 20% for UL54 part *1 and 2*, *UL56*, *UL89 exon 1 and 2, UL51,* and *UL27*. For UL97, the coefficient of variation was 35%. In fact, one SNP was detected in five passages and two SNPs in one passage. This additional SNP had a frequency of 19% and insufficient coverage.

#### Specificity

Detection specificity was evaluated by sequencing CMV genes in clinical samples positive for other *Herpesviridae* viruses and negative for CMV. The absence of reads corresponding to CMV in blood samples positive for HSV-1 and EBV viruses (6,742 copies/mL and 2,263 copies/mL respectively) confirmed the specificity of CHUGA NGS analysis. An *in silico* evaluation of primer sequence specificity was also performed.

Analytical specificity was 100%. In all tests conducted, no resistance mutations were erroneously detected compared to the reference results.

#### Accuracy of long-range PCRs and NGS assay

CMV gene (*UL97*, *UL54*, *UL56*, *UL89, UL51,* and *UL27*) sequencing results of NGS technology developed by CHUGA were compared with the reference Sanger technology for (i) 11 clinical samples from CHUGA patients presenting refractory CMV infection, of whom six had a nonsynonymous mutation conferring antiviral resistance; and (ii) five controls from QCMD, four of which had a nonsynonymous mutation conferring antiviral resistance (Table 3).

**TABLE 3:** Results of sequencing analysis of clinical samples from patients with refractory CMV infection and five controls from quality control for molecular diagnostics, obtained with CHUGA NGS technology and reference Sanger technology from CNR Herpesvirus Limoges^*a*^

Sample	Viral load (IU/mL)	Sanger sequencing (CNR Herpesvirus Limoges)		NGS sequencing (CHUGA)^*b*^			
		Mutation conferring resistance	Interpretation	Mutation conferring resistance	Frequency (%)	Read depth	Interpretation
1	2,814	*UL97*: no mutation *UL54*: no mutation *UL56*: C325Y *UL89*: NAF *UL27*: NAF *UL51*: NAF	LET resistance	*UL97*: NAF *UL54*: no mutation *UL56*: NAF *UL89*: no mutation *UL27*: NAF *UL51*: no mutation	–	–	–
2	11,143	*UL97*: M460I/V and C603C/W *UL54*: F412S *UL56*: NT *UL89*: no mutation *UL27:* no mutation *UL51:* no mutation	GCV resistance—detection of three strains; GCV-CDV resistances—detection of two strains	*UL97*: M460V/I and C603W *UL54*: F412S *UL56*: no mutation *UL89*: no mutation *UL27:* no mutation *UL51:* no mutation	45%, 12%, 27%, and 94%	279, 35, 268, and 873	GCV resistance—detection of three strains; GCV-CDV resistance—detection of two strains
3	2,601	*UL97*: no mutation *UL54*: A469T H686Y *UL56*: no mutation (P777L) *UL89*: no mutation *UL27:* no mutation *UL51:* no mutation	New mutations in the polymorphism area found in patients with resistance. Mutation in C terminal zone – no impact on LET	*UL97*: no mutation *UL54*: A469T H686Y *UL56*: no mutation (P777L) *UL89*: no mutation *UL27:* no mutation *UL51:* no mutation	85%, 95%, and 95%	438, 452, and 715	New mutations in polymorphism area found in patients with resistance
4	86,160	*UL97*: no mutation *UL54*: I568V *UL56*: NT *UL89*: no mutation *UL27:* no mutation *UL51:* no mutation	New mutation, without viral replication impact	UL 97: No mutation *UL54*: I568V *UL56*: no mutation *UL89*: no mutation *UL27:* no mutation *UL51:* no mutation	87%	518	New mutation, without viral replication impact
5	861,600	*UL97*: L595S *UL54*: no mutation *UL56*: NT *UL89*: NT	Dual population—sensitive/ resistant to ganciclovir	*UL97*: L595S 76%, 582 reads *UL54*: no mutation *UL56*: no mutation *UL89*: no mutation	76% and 63%	582 and 317	Dual population—sensitive/resistant to ganciclovir
6	157,400	*UL97*: T409M *UL54*: NAF *UL56*: NT	Resistant to MBV and sensitive to GCV	*UL97*: T409M *UL54*: no mutation *UL56*: no mutation	71%	437	Resistant to MBV and sensitive to GCV
7	3,953	*UL97*: no mutation *UL54*: no mutation *UL56*: no mutation *UL89*: NAF	–	*UL97*: NAF *UL54*: no mutation *UL56*: NT *UL89*: NAF	–	–	–
8	1,560	*UL97*: no mutation *UL54*: R542C *UL56*: NAF *UL89*: NAF	In the CNR evaluation—considered sensitive to GCV-FOS-CDV	*UL97*: no mutation *UL54*: R542C *UL56*: NAF *UL89*: NT	–	–	–
9	1,542	*UL97*: no mutation *UL54*: K513E E315D *UL89*: no mutation *UL27:* no mutation *UL51:* no mutation	GCV-CDV resistance increased replicative ability	*UL97*: no mutation *UL54*: K513E E315D *UL89*: no mutation *UL27:* NAF *UL51:* no mutation	61% and 95%	1,490 and 2,787	GCV-CDV resistance increased replicative ability
10	1,296	*UL97*: A591V *UL54*: K513E/K *UL56*: no mutation *UL89*: no mutation	Low-level resistance to ganciclovir dual populations – sensitive/resistant to GCV-CDV	*UL97*: A591V, *UL54*: K513E E315D *UL56*: no mutation *UL89*: no mutation	76%, 67%, and 95%	419, 339, and 921	Dual population—sensitive/resistant to GCV-CDV
11	15,222	*UL97*: no mutation *UL54*: no mutation (S290N) *UL56*: NT *UL89*: no mutation *UL27:* no mutation *UL51:* no mutation	*In vitro* S290R resistance to GCV and FOS (13)	*UL97*: no mutation *UL54*: no mutation (S290N) *UL56*: no mutation *UL89*: no mutation *UL27:* no mutation *UL51:* no mutation	62%	991	*In vitro* S290R resistance to GCV and FOS (13)
QCMD 1	4.01	*UL97*: no mutation *UL54*: no mutation	–	*UL97*: no mutation *UL54*: no mutation	–	–	–
QCMD 2	5.23	*UL54*: no mutation *UL97*: A594P	Low-level resistance to GCV	*UL97*: A594P *UL54*: no mutation	86%	8,374	Low-level resistance to GCV
QCMD 3	3.70	*UL97*: C603W *UL54*: no mutation	High-level resistance to GCV	*UL97*: C603W *UL54*: no mutation	96%	42,363	High-level resistance to GCV
QCMD 4	3.89	*UL97*: C603W *UL54*: no mutation	High-level resistance to GCV	*UL97*: C603W *UL54*: no mutation	95%	160	High-level resistance to GCV
QCMD 5	4.12	*UL97*: A594V *UL54*: A987G A834P	High-level resistance to CDV-FOS-GCV	*UL97*: A594V *UL54*: A987G A834P	93%, 91%, and 87%	19,005 65,332 63,378	High-level resistance to CDV-FOS-GCV

^*a*^ CDV: cidofovir; CHUGA: University Hospital Grenoble Alpes; CNR: national reference center; FOS: foscarnet; GCV: ganciclovir; LET: letermovir; MBV: maribavir; NAF: no amplification (samples not re-tested due to the absence of remaining DNA for additional assays); NT: not tested. Unwritten genes have not been tested due to insufficient samples.

^*b*^ – indicates no mutation conferring resistance was found.

All polymorphisms and resistance mutations detected with the reference Sanger technology were identified with CHUGA NGS ONT when testing and amplification were possible. The details of SNPs found for each technique are detailed in Table S4. Our pipeline accurately detected deletions (see QCMD 2 and 5 in Table S4). Some results were unavailable due to insufficient samples or lack of amplification (low viral loads). For a nucleotide polymorphism to be confirmed, the reading depth must exceed 100 reads/position. Double populations were well identified using this technology. The CHUGA NGS technology could amplify and sequence the CMV gene in samples with a viral load as low as 1,296 IU/mL (=sample 10) in which resistance to GCV and CDF occurred in a double population.

### Minor variants in mixed DNA from clinical samples

**Fig 2:**
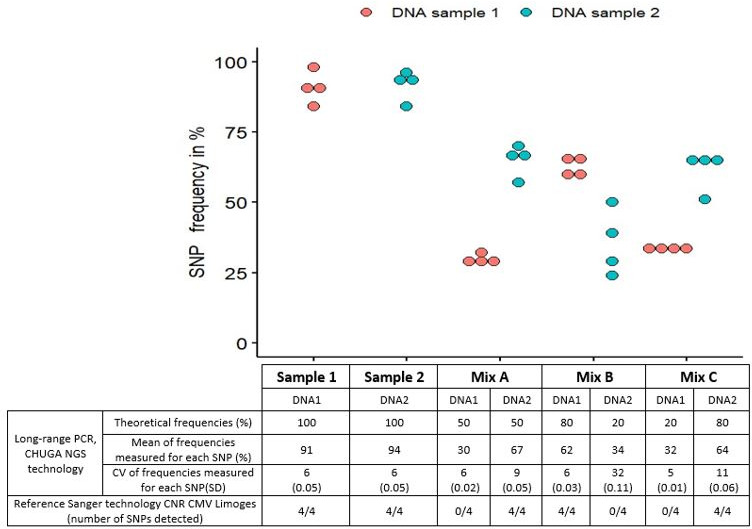
SNP detection frequencies on UL54 in mixed DNA using NGS technology compared with the reference Sanger technology. Red spots correspond to SNPs from sample 1 and blue spots to those from sample 2. CHUGA: University Hospital Grenoble Alpes; CV: coefficient of variability; PCR: polymerase chain reaction; SD: standard deviation; SNP: single-nucleotide polymorphism.

As illustrated in Fig. 2, sequencing of DNA1 and DNA2 showed that the CMV strain presented four specific SNPs in sample 1 (*UL54*: S676G – S897L – E1022D – E1152G) and four other specific SNPs in sample 2 (*UL54*: G629S – S655L – N685S – S1146G). Using the CHUGA NGS technology, all eight SNPs were detected in all mixes with a minimum of 1,500 read depths per amplicon. The measured frequency in each mix was similar to the expected theoretical frequency, except for DNA extracts at the 50% ratio. Indeed, the proposition obtained was 30% DNA1 and 67% DNA2. The coefficients of variation were all below 15%, except for mix B with a coefficient of variation of 32%. In this mix, measured frequencies of the four SNPs from sample 2 were heterogeneous, ranging from 24% to 39% when 20% was expected. Table S2 summarizes the frequencies and read depths. Any unexpected variants were detected.

Mixes were sequenced and analyzed with the Sanger reference technique (7, 29, 30). Primers are described in Table S3. In mixes A and C, only SNPs from sample 2 were detected. In mix B, only SNPs from sample 1 were detected.

### Detection threshold of minor variants in bacmid mixtures

Two bacmids were mixed in different ratios (100% to 2.5%). The CHUGA NGS technology detected mutation frequencies as low as 2.5% with a depth >1,000 reads, indicating a detection threshold below 2.5% (Table 4). Consistent with Alain et al., the reference Sanger technology did not detect mutations below 20% frequency (31). Mutations at frequencies of 5% or lower were not identified in the variant call table (VCF) after ClairS-TO but were visible in the Integrative Genomic Viewer.

**TABLE 4:** Detection frequency of resistance mutations in mixes of two bacmid DNA samples using NGS technology^*a*^^,*b*^

Sample	Bacmid ratio		Sanger reference technology CNR Herpesvirus		Long-range PCR and ClairS-TO, NGS technology, CHUGA	
	UL97 (E655D)	UL54 (N408S)	UL97 (E655D)	UL54 (N408S)	UL97 (E655D)	UL54 (N408S)
E1	20%	80%	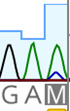	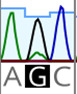	25%, 10,302 reads	70%, 11,497 reads
E2	10%	90%	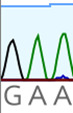	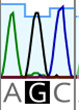	11%, 3,121 reads	84%, 10,337 reads
E3	5%	95%	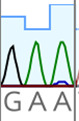	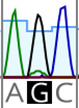	16%, 8,223 reads	80%, 10,296 reads
E4	2.5%	97.5%	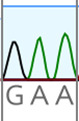	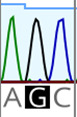	**5%, 7,575 reads**	90%, 8,346 reads
E5	100%		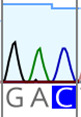	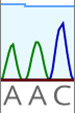	97%, 7736 reads	NA^*c*^
E6		100%	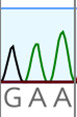	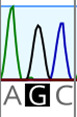	NA^*c*^	95%, 10,622 reads
E7	80%	20%	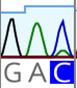	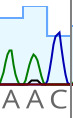	83%, 8,371 reads	14%, 6,539 reads
E8	90%	10%	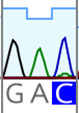	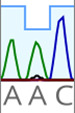	90%, 6,788 reads	6%, 6,592 reads
E9	95%	5%	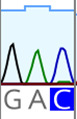	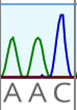	94%, 9,693 reads	**5%, 9,835 reads**
E10	97.5%	2.5%	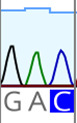	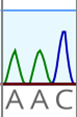	96%, 6,890 reads	**3%, 3,774 reads**

^*a*^ CHUGA, Grenoble-Alpes University hospital.

^*b*^ Bold indicates mutations not present in the variant call table but found on the integrated genome viewer.

^*c*^ NA, Not applicable.

### Double viral population detection with long-read sequencer

**Fig 3:**
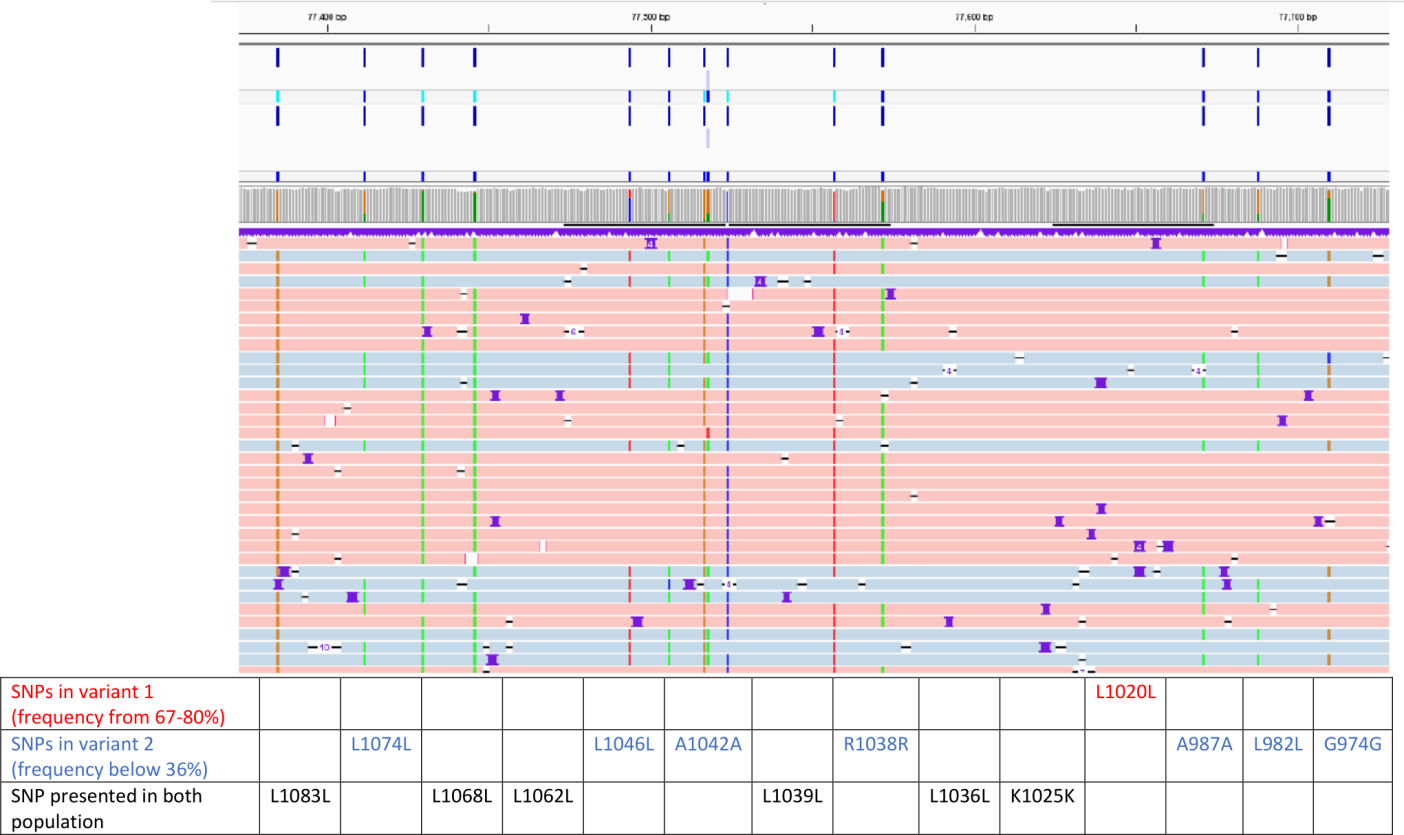
Visualization of the distribution of SNPs on UL54 raw sequencing data. Haplotype was determined by whatsHap (32) and visualized on IGV (33). Red reads indicate reads belonging to one subpopulation, while blue indicates a second subpopulation. SNPs presented in both populations is described in the table under the figure.

Using long-range PCR and ONT sequencing, the variant calling result was used to detect viral subpopulations with different variation frequencies identified on the sequenced amplicons. Mapped reads (Fig. 3) revealed two distinct CMV populations in a routine clinical sample, one at 75% and the other at 25%. Analysis of the UL54 gene identified 55 SNPs in the gene section. These SNPs allowed us to determine two sub-populations. Among these SNPs, five SNPs are related to a first variant (frequency distributions ranging from 67% to 80%, in red in the Table S5) and 11 SNPs are related to the second variant (frequency distributions ranging from 24% to 36%, in blue in the Table S4). The 39 remaining SNPs were natural polymorphism present in the two sub-populations (frequency distributions up to 87%). Similar frequency distributions were observed in *UL56*, *UL89*, and *UL97* (Table S4).

### NGS quality metrics

Quality metrics (Table 5) were based on the several tests described above, on supplier recommendations, and on our experience with the Oxford Nanopore technology used in our laboratory for several years. In addition to technical data, these quality metrics have taken into account the international clinical recommendations (15, 21, 34). Even if we were able to detect variants with less than 5% frequency, we decided not to systematically indicate it in our report to clinicians.

**TABLE 5:** Long-range PCRs followed by Oxford Nanopore sequencing quality metrics

Quality metrics for run validation	
PHRED quality score	More than 9
Positive control	All SNPs based on bottom criteria were detected
Negative control = extraction and amplification control	Less than 100 reads and no consensus on-target CMV
Quality metrics for sample validation	
Amplicon coverage	Minimum 80% of the amplicon with more than 100 reads^*a*^
Mapped reads per amplicon	More than 500 reads^*a*^
Quality metrics for SNP validation	
Read depth threshold	More than 100 reads^*a*^
Variant reporting frequency	5%^*b*^
Quality metrics for interpretation assignment per drug	
Based on mutation resistance database of CNR Herpesvirus Limoges	

^*a*^ Based on our sequencing experience and Oxford Nanopore recommendations.

^*b*^ Based on mixed bacmid experiments and clinical recommendations.

All validated SNPs located in a critical gene region and not matched in the database of CNR will be investigated at CNR Herpesvirus Limoges by phenotyping experiments.

## DISCUSSION

In this study, we developed eight long-range PCRs for sequencing the entire *UL54*, *UL97*, *UL27*, *UL89*, *UL56*, and *UL51* genes in a single run. This new technology was compared with the reference Sanger assay validated by the French CNR Herpesviruses Limoges. Sensitivity for viral load up to 2,600 IU/mL was ~90% for *UL54*, *UL51*, and *UL27* and ~70% for *UL89*, *UL97*, and *UL56* (Table 2), with *UL89* exon 1 showing the lowest sensitivity. This genome region is conserved in NCBI genomes, which does not explain this lower performance. Performance sensitivity for samples with viral load below 2,600 UI/mL appears lower than in other studies using short amplicons (24), although antiviral resistance testing is overwhelmingly prescribed for samples with viral loads > 2,600 UI/mL (15, 35, 36). Long-range PCR offers insights into viral dynamics and diversity (31), helping identify multiple populations within a sample (Fig. 3) (37). To detect minor variants in mixed DNA from clinical samples, the difference between measured and expected frequencies could be due to experimental variability as the proportions of extracted DNA were adjusted by adaptations to the volume and not by an exact quantification of the genetic material.

Concerning the wet-lab validation, long-range PCR of *UL97* and *UL54* was initially performed using previously published primers (34). Different conditions in annealing temperatures, primers, and DNA concentrations were tested on various samples (viral loads from 75,561 to 248,170 IU/mL) without satisfactory amplification results. Amplification of targets above 3,000 bp might be compromised due to the presence of fragmented DNA, especially in samples with low viral loads (38). This may lead to the design of new primers to amplify half genes above 3,000 bp. The amplification step could be seen as an obstacle to developing whole-genome sequencing, like preferential priming of one subpopulation over another. Although, contrary to shotgun analysis or other enrichment assays, our technology using only specific PCR amplification avoids sequencing bias (39).

The use of the rapid barcoding kit saved considerable time compared with the use of the standard ONT protocol. Sequencing equipment in biology laboratories makes the implementation of this technology easier and cheaper. The possibility of reusing a sequencing flowcell for several analyses after a simple wash reduces costs. The flowcell can be reused if at least 600 pores remain. Sequencing on a flongle was tested, but mapped reads and amplicon coverage were below sequencing on the flowcell, particularly with low viral load samples. Sequencing cost, including extraction and PCR, was evaluated at 200 €/sample for our technology and 150 €/sample with Sanger analysis. Time saving through bioinformatics is also an important point to consider: 1.5 days compared with 3 days using Sanger technology. The all bioinformatics management requires skilled staff and high-performance hardware and software to analyze and store large amounts of NGS data.

Dry-lab workflow was developed and adapted from viralrecon. Clair3 variant calling failed to list all mutations with frequencies below 20% (40) despite modifications in analysis parameters. With ClairS-TO, a deep learning method for long-read somatic small variant calling, mutations with frequencies between 20% and 2.5% were referenced in the VCF. Compared with Sanger analysis, our technology better detects low-frequency mutations and offers earlier detection of emerging resistant populations, leading to closer monitoring of these patients and, if necessary, a faster adaptation of antiviral treatments (21, 37, 41). Thanks to improvements in chemistry and basecalling algorithms, the error rate and detection of in-frame deletion mutations were improved (42). This new NGS assay can also detect multiple CMV subpopulations in CMV-infected patients. Some short-read sequencing data have demonstrated the within-host nucleotide diversity of CMV strains, suggesting frequent viral recombination (43). Estimating this diversity is difficult using amplicon sequencing but can be facilitated using long reads. Moreover, this technology can be used to study the genetic diversity of CMV from clinical isolates for epidemiological surveillance and to determine the strain landscape of circulating CMV (44). Indeed, one study showed that the viral load and time for CMV elimination could depend on the genotype (45). Thanks to these new diagnostic capabilities, it would also be interesting to assess whether early detection of resistance mutations can have an impact on patient management, and also on current recommendations for resistance CMV testing. It will be interesting to reassess these data in future multicenter clinical trials, including patients with persistent viremia under treatment.

Quality metrics of sequencing assays should be performed as resistant mutations may be missed due to low sensitivity, and on the contrary, false-positive mutation read-outs may occur, leading to the mistaken switching of therapy. Reliable testing is needed, and laboratories must have a clear understanding of the bioinformatic process in place (37). Given all the possible sequencing technologies (Illumina, Ion Torrent, ONT) and the different bioinformatics pipelines, resource sharing will facilitate standardization and assay development across different labs. Finally, despite the higher sensitivity and rapidity of NGS compared with Sanger, both approaches detected unknown SNPs that should be tested with phenotypic assays in the CNR Herpesvirus Limoges.

### Conclusion

We developed and validated long-range PCR of the six entire genes involved in CMV antiviral resistance, followed by NGS sequencing in a single run (ONT). This technology requires expertise in molecular biology and bioinformatics. To optimize these technologies, we recommend using a standardized mutation resistance database that is regularly updated and available online, as proposed by CNR Herpesvirus Limoges.

## Data Availability

The sequences are deposited in NCBI GenBank under PV630724-PV630776. Pipeline data are also available on the Github platform (https://gitlab.com/hugo.jardin2.0/cmv_drm). If readers need further information, they can contact the corresponding authors.

## SUPPLEMENTAL MATERIAL

Supplemental tables aac.00141-25-s0001.docx Tables S1 to S4.

Table S5 aac.00141-25-s0002.xlsx Variant caller file with double population.

ASM does not own the copyrights to Supplemental Material that may be linked to, or accessed through, an article. The authors have granted ASM a non-exclusive, world-wide license to publish the Supplemental Material files. Please contact the corresponding author directly for reuse.
