# Supplementary material for: Long-range PCRs and next-generation sequencing to detect cytomegalovirus drug resistance-associated mutations
Source: Antimicrob Agents Chemother. 2025 Jun 25;69(8):e00141-25. doi: 10.1128/aac.00141-25 (PMC12326992; doi:10.1128/aac.00141-25)
Supplement: Supplemental tables — Tables S1 to S4. [file aac.00141-25-s0001.docx]

**Supplementary data**

Supplementary Table 1: Rescue primer sequences (forward and reverse) designed in back-up for long-range polymerase chain reactions of cytomegalovirus (CMV) genes. Primers for UL27 are from CNR Limoges.

| Gene target | SEQUENCE PRIMER (5’->3’) | REFERENCE POSITION* | PRODUCT LENGTH (BP) |
| --- | --- | --- | --- |
| *UL54* Part 1 | CCTACGCAATACGGTCTCCC | 76699-76718 | 2500 |
|  | ATATTGCGGGTTCGGTGGTT | 79183-79202 |  |
| *UL54* Part 2 | CTGTATACGTCGGCAGGGTC | 78377-78396 | 2810 |
|  | CTATACTCGACAGCGGCGTT | 81164-81183 |  |
| *UL97* | TATAGCAATCCCCGTCACGC | 140368-140387 | 2376 |
|  | GCTCCTCATCGTCGTCGTAG | 142724-142743 |  |
| *UL56* | AAGATGAGGAAACCGCAGCA | 83414-83433 | 3081 |
|  | CGCTTTTCAGACCGCAACAA | 86474-86493 |  |
| *UL89* exon 1 | AGTTTTACGCGCCTGGGTTA | 132380-132399 | 2410 |
|  | GCACCAGGTGTAGAGGCAAT | 134770-134789 |  |
| *UL89* exon 2 | GAACGGACTTGGTACGCTGA | 136118-136137 | 2488 |
|  | GAGAGGCGGAGGAAGATTCG | 138585-138604 |  |
| *UL27* | TTCACGTACTGCTGTACGGTACG | 34973-34995 | 2476 (CNR Herpesvirus)^**^ |
|  | AGTTCTCACGGCTGATCTCG | 32478-32497 |  |
| *UL51* | AAAACAGCAGAGGGAGACGG | 73005-73024 | 1094 |
|  | GGCGGGGTGTTGAGGATTTA | 74079-74098 |  |

*CMV strain AD169 NCBI accession n° X17403.1

** The optimum Tm is modified to 62.5°C for this primer.

Supplementary Table 2: Mixed samples analysis. Description of frequencies (%) and read depths of each discriminant single nucleotide polymorphism (SNP) taken from the two samples and in each mix.

|  |  |  | Mix A | | Mix B | | Mix C | |
| --- | --- | --- | --- | --- | --- | --- | --- | --- |
|  | Sample 1 | Sample 2 | Sample 1 | Sample 2 | Sample 1 | Sample 2 | Sample 1 | Sample 2 |
| Theoretical frequencies (%) | 100 | 100 | 50 | 50 | 80 | 20 | 20 | 80 |
| SNP 1 (E1152G) | 84, 8044 |  | 28, 2532 |  | 60, 9084 |  | 32, 7988 |  |
| SNP 2 (E1022D) | 98, 8216 |  | 32, 8141 |  | 67, 8220 |  | 35, 8368 |  |
| SNP 3 (S897L) | 89, 7091 |  | 30, 7362 |  | 60, 7438 |  | 32, 7508 |  |
| SNP 4 (S676G) | 92, 4960 |  | 29, 5181 |  | 64, 5124 |  | 32, 5141 |  |
| SNP 5 (S1146G) |  | 84, 7657 |  | 57, 9026 |  | 24, 9047 |  | 51, 8175 |
| SNP 6 (N685S) |  | 95, 5197 |  | 66, 5214 |  | 29, 5161 |  | 64, 5388 |
| SNP 7 (S655L) |  | 92, 4952 |  | 67, 4826 |  | 29, 4623 |  | 64, 5044 |
| SNP 8 (G629S) |  | 96, 4768 |  | 70, 4282 |  | 27, 4073 |  | 66, 4522 |

Supplementary Table 3: Rescue primers sequences (forward and reverse) used for Sanger analysis of cytomegalovirus (CMV) genes in national reference center Herpervirus Limoges.

| Gene target | SEQUENCE PRIMER (5’->3’) | REFERENCE POSITION* | PRODUCT LENGTH (BP) |
| --- | --- | --- | --- |
| *UL54* External | CGGCAACAACCTATACGAG | 76,667-76,685 | 4130 |
|  | CTCCGACGAAGAAGASAACG | 80,777-80796 |  |
| *UL54* Internal | GTCTCAGCAGCATCATCACC | 76,853-76,872 | 3814 |
|  | TTGCTTCGTAAGCTGTCAGC | 80,647-80,666 |  |
| *UL97* External | CCGATACAGTCAGCGAACG | 140,135-140,153 | 2727 |
|  | TAAGACAGACGCAGCACCG | 142,843-142,861 |  |
| *UL97* Internal | ATAGCAATCTCCGTCACGCC | 140,369-140,388 | 2373 |
|  | TCCTCATCGTCGTCGTAGTCC | 142,721-142,741 |  |
| *UL56* External | CGCGTCGCTGATGGACAAGT | 86,921-86,940 | 4112 |
|  | GGGTGTTGGAATAATCGTCG | 82,829-82,848 |  |
| *UL56* Internal | GCGAGTTATTTGTGCACCG | 86,071-86,089 | 2924 |
|  | GATATTACGTTCAAAGCGAA | 83,166-83,185 |  |
| *UL89*-A External | AGCGATCGTAAGGTGACACG | 138,795-138,814 | 1690 |
|  | CCATGACGCAGTCGCTCAT | 137,125-137,143 |  |
| *UL89*-A  Internal | ACGAAGACGAACGAGTGACG | 138,525-138,544 | 1185 |
|  | CTGTCCGGTGCTTAAGAACC | 137,360-137,379 |  |
| *UL89*-B External | GGATGTCGTTAACGCACTCC | 134,008-134,027 | 1847 |
|  | TTTGTTGCTCAATTCGCTGCA | 132,181-132,201 |  |
| *UL89*-B  Internal | TAGGCTTCCAGTCGAGCGT | 133,642-133,660 | 1480 |
|  | TTTGTTGCTCAATTCGCTGCA | 132,181-132,201 |  |
| UL27 External | TTCACGTACTGCTGTACGGTACG | 34,973-34,995 | 2476 |
|  | AGTTCTCACGGCTGATCTCG | 32,478-32,497 |  |
| *UL27* Internal | CGTGAAGGAGGAGACTGTGA | 34,739-34,758 | 2155 |
|  | CCACCAGCAGCTTCCAGTAT | 32,604-32,623 |  |

*CMV strain AD169 NCBI accession n° X17403.1

Supplementary Table 4: Details of the SNPs found in the clinical samples and controls from Quality Control for Molecular Diagnostics, obtained with CHUGA NGS technology and reference Sanger technology from CNR Herpesvirus. SNPs detected in only one of the 2 techniques are noticed in brackets with their frequency if < 40%)

| **Sample** | **Viral load (IU/mL)** | **Technique for sequencing** | **Number of SNPs** | | | | | | |
| --- | --- | --- | --- | --- | --- | --- | --- | --- | --- |
|  |  |  | **UL97** | **UL54** | **UL56** | **UL89** | **UL27** | **UL51** |  |
| 1 | 2,814 | **Sanger** | 0 | 42 (+ I409I) | 26 | NAF | NAF | NAF |  |
|  |  | **NGS** | NAF | 42 (+ S897S + L1039L) | NAF | 0 | NAF | 0 |  |
| **2** | 11,143 | **Sanger** | 20 (+ G598G) | 47 | NT | 4 | 28 | 2 |  |
|  |  | **NGS** | 20 (+ N68D + N68N + I244V) | 47 (+ R1052C + G1208D) | 30 | 4 | 28 | 2 |  |
| **3** | 2,601 | **Sanger** | 15 | 41 | 27 | 3 | 21 (+ L324L + F545F + L554L) | 4 |  |
|  |  | **NGS** | 15 (+ N68D + I244V) | 41 (+ A1164A) | 27 | 3 | 19(+ G570G) | 4 |  |
| **4** | 86,160 | **Sanger** | 15 | 43 | NT | 14 | 15 | 4 |  |
|  |  | **NGS** | 15 (+ N68D + L126Q + I244V) | 43 (+ S897S) | 28 | 14 | 15 | 4 |  |
| **5** | 861,600 | **Sanger** | 16 | 52 | NT | NT | NT | NT |  |
|  |  | **NGS** | 16 (+ N68D + L126Q + I244V + A594V (17%) + L595S) | 52 | 26 | 0 | NT | NT |  |
| **6** | 157,400 | **Sanger** | 17 | NAF | NT | NT | NT | NT |  |
|  |  | **NGS** | 17 (+ 5N68D + I244V + H411Y (17%)) | 52 | 26 | NT | NT | NT |  |
| **7** | 3,953 | **Sanger** | 15 | 52 | 0 | NAF | NT | NT |  |
|  |  | **NGS** | NAF | 52 | NT | NAF | NT | NT |  |
| **8** | 1,560 | **Sanger** | 18 | 67 (+ F443F) | NAF | NAF | NT | NT |  |
|  |  | **NGS** | 18 (+ N68D + I244V) | 67 | NAF | NT | NT | NT |  |
| **9** | 1,542 | **Sanger** | 16 | 53 | NT | 17 | 25 | 3 |  |
|  |  | **NGS** | 16 (+ N68D + L126Q + I244V + A497S + N510S) | 53 | NT | 17 | NAF | 3 |  |
| **10** | 1,296 | **Sanger** | 21 | 50 (+ S623S + E1145E) | 29 | 17 (+ N296K) | NT | NT |  |
|  |  | **NGS** | 21 | 50 (+ E315D + S517S) | 29 (+ R369S) | 17 | NT | NT |  |
| **11** | 15,222 | **Sanger** | 17 | 53 (+ S623S) | NT | 10 | 22 (+ P543P) | 5 (+ T47T + A44A + H65H/R + D71E/D) |  |
|  |  | **NGS** | 17 (+ T154T + R433R (12%)) | 53 (+ 13 SNPs with frequency < 40%) | 30 | 10 | 22 (+ P7L + T13T + E22E + L75L + T128T + C210C + V446V) | 5 |  |
| **QCMD 1** | 4.01 | **Sanger** | 21 | 44 | NT | NT | NT | NT |  |
|  |  | **NGS** | 21 | 44 | NT | NT | NT | NT |  |
| **QCMD 2** | 5.23 | **Sanger** | 2 | 25 + del D981_L982 | NT | NT | NT | NT |  |
|  |  | **NGS** | 2 | 25 + del D981_L982 | NT | NT | NT | NT |  |
| **QCMD 3** | 3.70 | **Sanger** | 12 | 58 | NT | NT | NT | NT |  |
|  |  | **NGS** | 12 | 58 (+ G1027D (7%)) | NT | NT | NT | NT |  |
| **QCMD 4** | 3.89 | **Sanger** | 20 | 40 | NT | NT | NT | NT |  |
|  |  | **NGS** | 20 | 40 | NT | NT | NT | NT |  |
| **QCMD 5** | 4.12 | **Sanger** | 20 | 46 + del G1151 | NT | NT | NT | NT |  |
|  |  | **NGS** | 20 | 46 (+ S894L) + del G1151 | NT | NT | NT | NT |  |

*SNP: Single nucleotide polymorphism. Sanger was performed in CNR Herpesviridae in Limoges; NGS corresponding to our technique at the University Hospital Grenoble Alpes; NAF: no amplification (samples not re-tested due to the absence of remaining DNA for additional assays); del: deletion; NT: not tested*

Supplementary Table 5: Variant call table showing the frequency and distribution of single nucleotide polymorphisms on different genes using long-read polymerase chain reactions.

See excel file
